# Stress Wave Propagation in Viscoelastic-Plastic Rock-Like Materials

**DOI:** 10.3390/ma9050377

**Published:** 2016-05-17

**Authors:** Liu Lang, KI-IL Song, Yue Zhai, Dezheng Lao, Hang-Lo Lee

**Affiliations:** 1 Energy School, Xi’an University of Science and Technology, Xi’an 710054, China; csuliulang@163.com; 2 Key Laboratory of Western Mines and Hazards Prevention, Ministry of Education of China, Xi’an 710054, China; 3 Department of Civil Engineering, Inha University, Incheon 402-751, Korea; dwighthaward@naver.com; 4 School of Geology Engineering and Geomatics, Changan University, Xi’an 710054, China; zy@chd.edu.cn; 5 School of Civil & Resource Engineering, University of Western Australia, Perth 6009, Australia; spring_xue@163.com

**Keywords:** viscoelastic-plastic rock-like materials, constitutive equation, stress wave equation, strain rate, attenuation, SHPB apparatus

## Abstract

Rock-like materials are composites that can be regarded as a mixture composed of elastic, plastic, and viscous components. They exhibit viscoelastic-plastic behavior under a high-strain-rate loading according to element model theory. This paper presents an analytical solution for stress wave propagation in viscoelastic-plastic rock-like materials under a high-strain-rate loading and verifies the solution through an experimental test. A constitutive equation of viscoelastic-plastic rock-like materials was first established, and then kinematic and kinetic equations were then solved to derive the analytic solution for stress wave propagation in viscoelastic-plastic rock-like materials. An experimental test using the SHPB (Split Hopkinson Pressure Bar) for a concrete specimen was conducted to obtain a stress-strain curve under a high-strain-rate loading. Inverse analysis based on differential evolution was conducted to estimate undetermined variables for constitutive equations. Finally, the relationship between the attenuation factor and the strain rate in viscoelastic-plastic rock-like materials was investigated. According to the results, the frequency of the stress wave, viscosity coefficient, modulus of elasticity, and density play dominant roles in the attenuation of the stress wave. The attenuation decreases with increasing strain rate, demonstrating strongly strain-dependent attenuation in viscoelastic-plastic rock-like materials.

## 1. Introduction

Previous studies have examined the propagation of the stress wave in composite materials, such as rock and concrete under a loading for the seismic design of various infrastructure systems [1,2,3].

The propagation of the stress wave is governed mainly by the inherent physical properties of the material and characteristics of the stress wave. Although brittle rock-like materials (e.g., fresh granite) are typically heterogeneous and show brittle fracture behaviors under a static load and low-strain-rate load, their dynamic behaviors are dependent on the strain rate under a high-strain-rate load [4,5,6]. In general, mechanical responses of the materials dependent on the strain rate can be divided into two categories: time-independent transient responses (responses not dependent on the strain rate) and time-related non-transient responses (responses dependent on the strain rate) [7]. From a macroscopic perspective, the dispersion phenomenon is equivalent to the absorption phenomenon in that the two phenomena are commonly induced by the dissipative force of viscous materials from the viscosity effects.

An analytical solution for stress wave propagation in viscoelastic materials is used widely in polymer materials based on the viscosity effects according to the elastic responses [8]. An analytical solution for stress wave propagation in viscoplastic materials has been developed by considering the viscosity effects based on the plastic responses [9]. A theoretical solution for stress wave propagation in viscoelastic-plastic materials that considers the viscosity effects on both elastic and plastic parts has been developed based on the elastic-plastic responses [10].

## 2. Analytic Solution for Stress Wave Propagation in Viscoelastic-Plastic Rock-Like Materials

### 2.1. Constitutive Equation of Viscoelastic-Plastic Rock-Like Materials

To derive a constitutive equation for viscoelastic-plastic rock-like materials under a high-strain-rate load, such a material should be assumed to be a composite material composed of elastic, plastic, and viscous components. In addition, these three components should be assumed to be distributed randomly and that the mixture reflects the macroscopic dynamic and mechanical behaviors of viscoelastic-plastic rock-like materials. According to element model theory [11,12,13], which is widely accepted in the constitutive equation of rock-like materials, the constitutive model of viscoelastic-plastic rock-like materials can be established.

**Figure 1:**
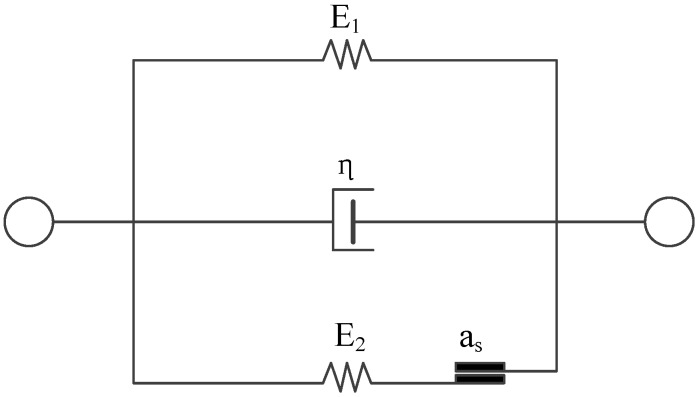
Configuration of the constitutive model for viscoelastic-plastic rock-like materials. Note: a_s_ is St. Venant element.

This model is composed of three main components: an elastomer, a Newtonian body, and a St. Venant body. Figure 1 shows the constitutive model of viscoelastic-plastic rock-like materials, and the constitutive equations are presented as follows:

(1) Elastomer. The stress–strain relationship is linear elastic. The constitutive equation is:

(1) $$\sigma_{E}=E_{1}\varepsilon_{E}$$

where *E*_1_ is the initial elastic modulus of the elastic component, ε*_E_* and σ*_E_* are the strain and stress of the elastomer, respectively.

(2) Newtonian body. The stress is proportional to the strain; there is no immediate deformation, and the constitutive equation can be defined as:

(2) $$\sigma_{N}=\eta {\dot{\varepsilon }}_{N}$$

where η is the viscosity coefficient, ${\dot{\varepsilon }}_{N}$ and σ*_N_* are the strain rate and stress of the Newtonian body, respectively.

(3) St. Venant body. To be ideal elastic-plastic, there should be no creep, relaxation, and springback. The constitutive equation is:

(3a) $$\sigma_{SV}=E_{2}\varepsilon_{SV},\;\sigma_{SV}<\sigma_{S}$$

(3b) $$\varepsilon_{S}\to \infty ,\;\sigma_{SV}\geq \sigma_{S}$$

where *E*_2_ is the initial elastic modulus of the St. Venant body and σ*_S_* is the elastic limit. The elastic limit is the lowest stress level at which the material begins to deform plastically. The elastic limit should be determined experimentally. If the stress on the specimen exceeds the elastic limit, the stress-strain curve becomes nonlinear because of the plastic strain component [2].

According to element model theory and the parallel relationship between three stress components, the stress and strain should satisfy the following:

(4a) $$\sigma =\sigma_{E}+\sigma_{SV}+\sigma_{N}$$

(4b) $$\varepsilon =\varepsilon_{E}=\varepsilon_{SV}=\varepsilon_{N}$$

From the three sets of constitutive equations of the model elements and their parallel relationship, differential equations of the constitutive model for viscoelastic-plastic rock-like materials can be derived as:

(5a) $$\sigma =\left(E_{0}+E_{2}\right)\varepsilon +\eta \left(\frac{E_{0}+E_{1}+E_{2}}{E_{1}}\right)\dot{\varepsilon }-\frac{\eta \dot{\sigma }}{E_{1}},\;\sigma <\sigma_{S}$$

(5b) $$\sigma =E_{0}\varepsilon +\eta \left(\frac{E_{0}+E_{1}}{E_{1}}\right)\dot{\varepsilon }-\frac{\eta \dot{\sigma }}{E_{1}}+E_{2}\varepsilon_{S},\;\sigma \geq \sigma_{S}$$

Equation (5) is a special case of the constitutive equations for viscoelastic-plastic rock-like materials and can be rewritten in a general form as follows:

(6) $$\alpha_{0}\sigma +\alpha_{0}\dot{\sigma }=b_{0}\varepsilon +b_{10}\dot{\varepsilon }$$

This equation is called a standard linear solid model. To describe the general behaviors of viscoelastic-plastic rock-like materials, a generalized linear viscoelastic-plastic model should be defined as:

(7) $$\overset{m}{\underset{i=0}{\sum }}a_{i}\frac{\partial^{i}\sigma }{\partial t^{i}}=\overset{n}{\underset{i=0}{\sum }}b_{i}\frac{\partial^{i}\varepsilon }{\partial t^{i}}$$

where *a_i_* and *b_i_* are the material parameters.

### 2.2. The Wave Equation of Viscoelastic-Plastic Rock-Like Materials

This study confines stress wave propagation along the slender bar. Stress wave propagation along the slender bar can be simulated experimentally using an SHPB device. Through this test, the dynamic properties of a specimen material can be obtained [14,15,16,17]. The fundamental theory of the stress wave propagation in a slender bar can also be used to test the pile integrity.

To establish a stress wave equation for viscoelastic-plastic materials, kinematical and kinetic equations are incorporated with a constitutive equation of viscoelastic-plastic rock-like materials [18]. In the coordinate system, the central axis of a slender rock bar is taken as the *x*-axis, and the length is set to far exceed the diameter of the bar. Basically, $A_{0}$ (the initial cross-sectional area prior to deformation), $\rho_{0}$ (initial density), and the material parameters have no relationships with the coordinate system. In the remainder of this paper, *u* (displacement of the material point), ε (strain), ν (particle velocity), and σ (stress) are the longitudinal components along the *x*-axis.

The kinematic equation is often called a continuity equation or a mass conservation equation. A compatibility equation about ε and *u* can be established based on the monotropic continuity condition of displacement *u*:

(8) $$\frac{\partial u}{\partial X}=\frac{\partial \varepsilon }{\partial t}$$

The kinetic equation is called an equation of motion or a momentum conservation equation. The following equation can be established based on the continuity condition as follows:

(9) $$\rho_{0}\frac{\partial v}{\partial t}=\frac{\partial \sigma }{\partial X}$$

A constitutive equation (physical equation) presents a continuous function between strain and stress. A piecewise continuous function can also be used in a constitutive equation in a manner similar to that in Equation (5).

Because the velocity of the stress wave induced by impact loading in hard rock materials is high and the travel time in the microelement is too short to cause heat exchange with the neighboring microelement, the process of impact loading can be considered to be approximately adiabatic. Therefore, it is unnecessary to implement an energy conservation equation to derive a closed-form solution for σ, ε, and *ν*.

If viscoelastic-plastic rock-like materials are subject to a high-strain-rate compressive loading, then the stress wave is fully reflected in the fractured surface formed by the damage induced by the shock wave. Therefore, the stress wave cannot propagate forward. To ensure the stress function σ(ε) as a continuous differentiable function, this paper focuses mainly on the mechanical properties of viscoelastic-plastic rock-like materials without cracks. Under such a condition, the stress does not exceed the yield stress. Therefore, $E_{d}=d\sigma /d\varepsilon \geq 0$, and the stress wave velocity can be defined as follows:

(10) $$C=\pm \sqrt{\frac{1}{\rho_{0}}\frac{d\sigma }{d\varepsilon }}=\sqrt{\frac{1}{\rho_{0}}E_{d}}$$

where *C* is the stress wave front velocity. The sine stress is a monotropic function of the strain; *C* is a monotropic function of strain; and $\rho_{0}C$ is the impedance of the material, which plays a decisive role in stress wave propagation. In Equation (10), the positive sign corresponds to the wave traveling from right to left, and *vice versa* for the negative sign.

The stress wave equation for viscoelastic-plastic rock-like materials can be obtained by solving kinematic and kinetic equations (Equations (8) and (9), respectively) and the constitutive equation (Equation (5)) for viscoelastic-plastic rock-like materials. If *u* (displacement) is considered to be an unknown quantity, then the calculation process is as follows:

If $\sigma <\sigma_{s}$, then substitute $\varepsilon =\frac{\partial u}{\partial X}$ and $v=\frac{\partial u}{\partial t}$ into Equation (5a) separately and take the first derivative with respect to the *x*-axis on both the left and right sides:

(11) $$\frac{\partial \sigma }{\partial X}=\left(E_{0}+E_{2}\right)\frac{\partial^{2}u}{\partial X^{2}}+\eta \left(\frac{E_{0}+E_{1}+E_{2}}{E_{1}}\right)\frac{\partial^{3}u}{\partial t\partial X^{2}}-\frac{\eta \partial^{2}\sigma }{E_{1}\partial t\partial X}$$

Substituting Equation (7) into Equation (9) results in the following equation:

(12) $$\rho_{0}\frac{\partial^{2}u}{\partial t^{2}}=\left(E_{1}+E_{2}\right)\frac{\partial^{2}u}{\partial X^{2}}+\eta \frac{\partial^{3}u}{\partial t\partial X^{2'}}\;\sigma <\sigma_{s}$$

This equation is a third-order partial differential equation of *u*. If the travel time of the stress wave is short, then the viscosity term containing the third derivative can be ignored. Therefore, the above equation can be expressed as a second-order partial differential equation for stress wave propagation along the elastic bar.

Similarly,

(13) $$\rho_{0}\frac{\partial^{2}u}{\partial t^{2}}=E_{1}\frac{\partial^{2}u}{\partial X^{2}}+\eta \frac{\partial^{3}u}{\partial t\partial X^{2'}}\;\sigma \geq \sigma_{s}$$

Because of the viscosity term, the elastic wave equation $u=F\left(X+C_{0}t\right)$ does not satisfy the stress wave equation (Equation (14)). To solve this problem, a harmonic wave solution can be adopted:

(14) $$u\left(X,t\right)=A\;\cdot \;\text{exp}\left[i\left(\omega t-k_{i}X\right)\right]$$

Substituting Equation (14) into Equation (12) yields:

(15) $$\rho_{0}\omega^{2}-\left(E_{1}+E_{2}\right)k_{i}^{2}-\eta i\omega k_{i}^{2}=0$$

Because this is an equation with a complex number, the frequency ω can be considered a real number, and $k_{i}$ must be a complex number:

(16) $$k_{i}=k+i\alpha$$

Substituting Equation (16) into Equation (15) yields:

(17) $$\rho_{0}\omega^{2}-\left(E_{1}+E_{2}\right)\left(k^{2}-\alpha^{2}\right)-2ki\alpha \left(E_{1}+E_{2}\right)-\eta i\omega \left(k^{2}-\alpha^{2}\right)+2\eta \alpha k\omega =0$$

If the real component is set to be equal to the imaginary component, then the following equations can be derived:

(18) $$\rho_{0}\omega^{2}-\left(E_{1}+E_{2}\right)\left(k^{2}-\alpha^{2}\right)+2\eta \alpha k\omega =0\;$$

(19) $$\left(E_{1}+E_{2}\right)2k\alpha +\eta \omega \left(k^{2}-\alpha^{2}\right)=0$$

Solving Equations (18) and (19) with respect to the attenuation factor α and the wave number *k* gives the following:

(20a) $$\alpha^{2}=\frac{\rho_{0}\omega^{2}\sqrt{{\left(E_{1}+E_{2}\right)}^{2}+\eta^{2}\omega^{2}}-\left(E_{1}+E_{2}\right)}{2\left[\eta^{2}\omega^{2}+{\left(E_{1}+E_{2}\right)}^{2}\right]}$$

(20b) $$k^{2}=\frac{\rho_{0}\omega^{2}\sqrt{{\left(E_{1}+E_{2}\right)}^{2}+\eta^{2}\omega^{2}}+\left(E_{1}+E_{2}\right)}{2\left[\eta^{2}\omega^{2}+{\left(E_{1}+E_{2}\right)}^{2}\right]}$$

The attenuation factor α can be either positive or negative. Because a positive root means that the amplitude increases with increasing propagation distance, it violates the law of energy conservation and has no physical meaning. Therefore, the attenuation factor α should be negative. The solution to Equation (12) can be obtained as follows:

(21) u(X,t)=Aexp(−αX)exp[i(ωt−kX)]

Similarly, if $\sigma \geq \sigma_{s}$, then the harmonic wave solution in Equation (14) is the solution to Equation (13), and its form is similar to Equation (20). Under this condition, the wave number *k* and the attenuation factor α can be determined, respectively, using the following equations:

(22a) $$\alpha^{2}=\frac{\rho_{0}\omega^{2}\left(\sqrt{E_{1}^{2}+\eta^{2}\omega^{2}}-E_{1}\right)}{2\left(E_{1}^{2}+\eta^{2}\omega^{2}\right)}$$

(22b) $$k^{2}=\frac{\rho_{0}\omega^{2}\left(\sqrt{E_{1}^{2}+\eta^{2}\omega^{2}}+E_{1}\right)}{2\left(E_{1}^{2}+\eta^{2}\omega^{2}\right)}$$

Equations (20) and (22) can be rewritten as:

(23a) $$\alpha^{2}=\frac{\rho_{0}\omega^{2}\left(\sqrt{b^{2}+1}-b\right)}{2\eta \left(b^{2}+1\right)}$$

(23b) $$k^{2}=\frac{\rho_{0}\omega^{2}\left(\sqrt{b^{2}+1}+b\right)}{2\eta \left(b^{2}+1\right)}$$

where $b=\frac{E}{\eta \omega }$, *E* is the nominal elastic modulus. Here, $E=E_{1}+E_{2}$ if $\sigma <\sigma_{s}$, and $E=E_{1}$ if $\sigma \geq \sigma_{s}$, respectively.

Therefore, the elastic modulus of the material decreases when the stress exceeds the plastic threshold. The stress wave is influenced significantly by the attenuation factor under such a condition.

The particle velocity and strain of materials can be defined using spatial and temporal positions:

(24) $$v\left(X,t\right)=\frac{\partial u\left(X,t\right)}{\partial t}=A\exp\left(-\alpha X\right)\exp\left[i\left(\omega t-kX\right)\right]i\omega =i\omega u\left(X,t\right)$$

(25) $$\varepsilon \left(X,t\right)=\frac{\partial u\left(X,t\right)}{\partial t}=-i\left(\alpha +k\right)A\exp\left(-\alpha X\right)\exp\left[i\left(\omega t-kX\right)\right]=-i\left(\alpha +k\right)u\left(X,t\right)$$

The attenuation speed depends on the attenuation factor of the stress wave α, and its unit is [dB/m]. From Equations (20a) and (22a), the analytical solution for stress wave propagation in viscoelastic-plastic rock-like materials suggests that the attenuation factor α can be determined by the viscous coefficient of the material, the elastic modulus, density, and the frequency of the stress wave.

## 3. Verification of the Analytic Solution for Stress Wave Propagation

### 3.1. An Experimental Test Using the SHPB and an Inverse Analysis

The mean strain rate $\dot{\varepsilon }$ and the elastic limit $\sigma_{s}$ in the constitutive equation, Equation (5) can be obtained experimentally. The elastic modulus (*E*_1_ and *E*_2_) and the viscosity coefficient η are parameters to be determined. To obtain these unknown parameters, an experimental test using the SHPB (Split Hopkinson Pressure Bar) was adopted. The SHPB is an experimental technology to examine the dynamic mechanical properties of engineering materials. The pressure rod is assumed to be a simple elastic rod. Owing to impact load at one end, the elastic stress waves are propagated and cause deformation (*i.e.*, elastic deformation and plastic deformation).

**Figure 2:**
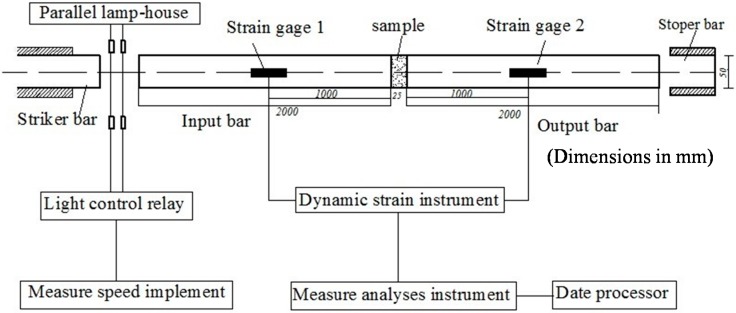
SHPB apparatus.

**Figure 3:**
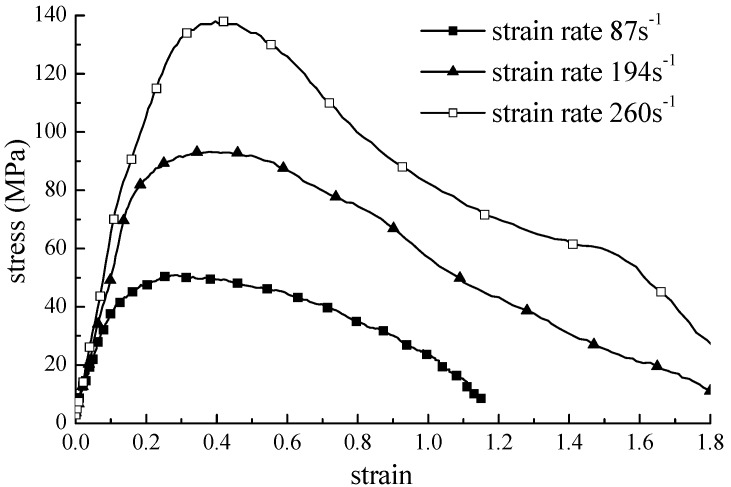
Stress-strain curve depending on the strain rate.

This study verified the analytic solution derived in the previous section by conducting an SHPB test with concrete samples composed of fine aggregates. The height and diameter of the sample were 25 mm and 50 mm, respectively. The density of the sample was 2080 kg·m^−3^, and the maximum grain size was 2.5 mm. Figure 2 presents the SHPB apparatus. Based on the SHPB test of viscoelastic-plastic materials, the effects of the viscous coefficient, the elastic modulus, density, and the frequency of the stress wave on the attenuation factor were investigated. The stress-strain curves for the concrete specimen for three strain rates are presented in Figure 3.

The elastic stress wave along the guide rod in the SHPB can be decomposed into a series of harmonic components using the spectrum analysis method. The frequency and wavelength of harmonics are different from each other, and the harmonics propagate at different phase velocities [18]. Because the wavelength of the stress wave depends on the bullet length, the frequency of the stress wave varies, even for the same strain rate [19]. Dynamic properties of materials were not yet verified to be related directly to the frequency of the stress wave. Therefore, the effect of the frequency was not considered in the present study for parameter identification purposes.

**Figure 4:**
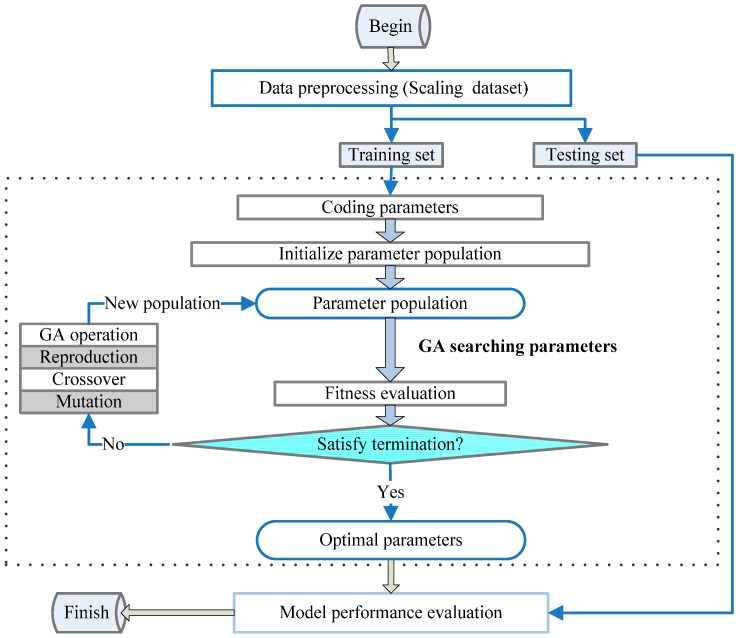
Schematic of the genetic algorithm (GA) used to determine parameters.

The identification process of unknown parameters using experimental test results is an optimization problem. An optimal solution providing the minimum mean square error in the objective function can be obtained as:

(26) $${\hat{\lambda }}^{2}=\overset{N-1}{\underset{i=1}{\sum }}\frac{{\left\{\left[\sigma \left(x\right)-\sigma_{i}\right]/\sigma_{i}\right\}}^{2}}{N-\text{num}}$$

where σ(x) is the calculated stress that can be obtained by strain ε*_i_* measured from an experimental test for the objective function. The unit of σ(*x*) is MPa; σ*_i_* is the stress for testing; num is the number of unknown parameters; and *N* is the number of experimental data observations. The optimization technique used in the present study is the genetic algorithm (GA), which can address complicated problems because it is based on the process of a biological evolution, rules governing the survival of the fittest, and the chromosome information exchange mechanism. The GA has various advantages and is well described [20,21], and Figure 4 presents a specific schematic of the GA. Table 1 lists the parameters obtained from an inverse analysis using the GA.

**Figure 5:**
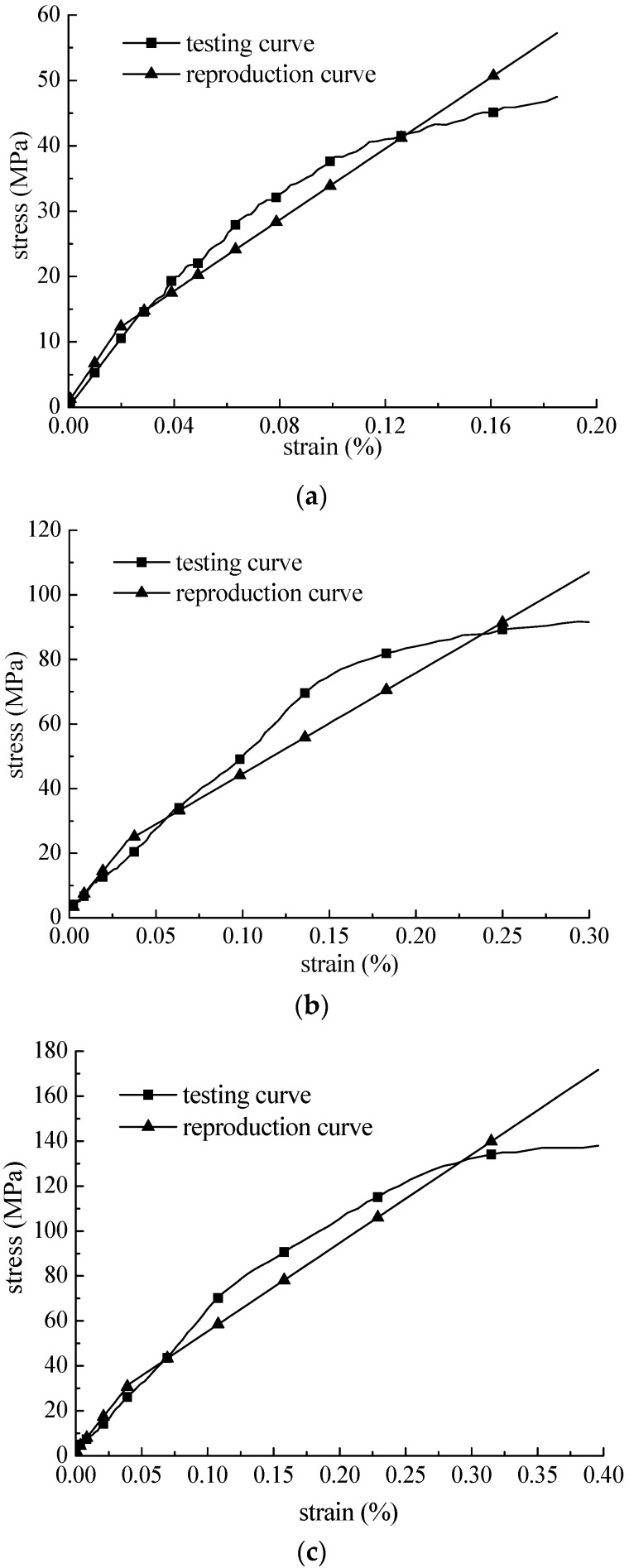
Viscoelastic-plastic dynamic stress-strain curves. (**a**) Strain rate: 87 s^−1^; (**b**) strain rate: 194 s^−1^; (**c**) strain rate: 260 s^−1^.

The stress-strain curves from the analytical solution were reproduced using the parameters obtained from inverse analysis. The test and reproduced curves are presented in Figure 5. The estimated stress-strain curve from the analytic solution was identical to the stress-strain curve from the experimental test regardless of the strain rate, and the mean square error between them was less than 10% of the strain range of the intersections. The newly derived constitutive equation and parameters from inverse analysis were validated in terms of their applicability and reliability.

### 3.2. Attenuation Factors in the Stress Wave Equation

The effects of the frequency of the stress wave on the attenuation in viscoelastic-plastic rock-like materials depends mainly on the imperfect elasticity (plasticity and viscosity). Previous studies have reported that microscopic factors are involved in this process, including the composition of the rock mineral, particle size, internal crack distribution, structures, and pore fluids [22]. The amplitude of attenuation is presented as:

(27) $$A\left(x\right)=A_{0}\exp\left(\alpha x\right)$$

where $A_{0}=A(x){|}_{x=0}$ is the strength of the dynamic impact source. The attenuation amplitude of the propagating stress wave increases exponentially with increasing propagation distance, and the extent of attenuation is determined by the attenuation factor α.

**Figure 6:**
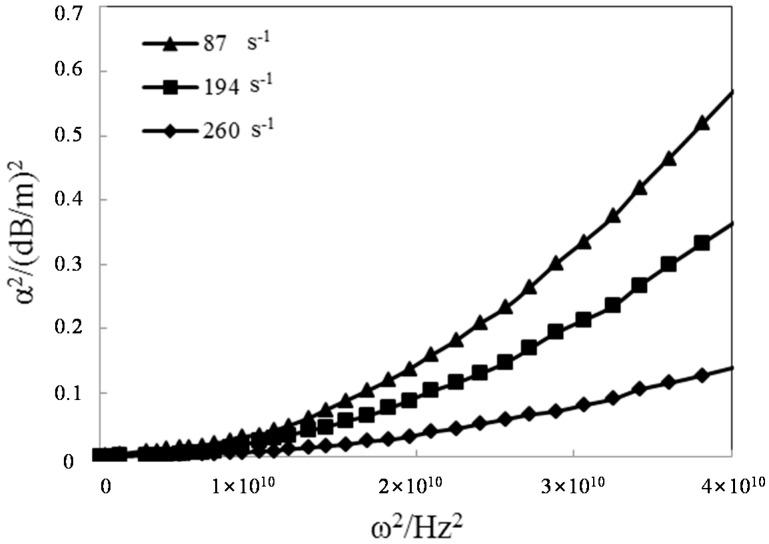
Effects of the stress wave frequency on attenuation factors in viscoelasticity.

**Figure 7:**
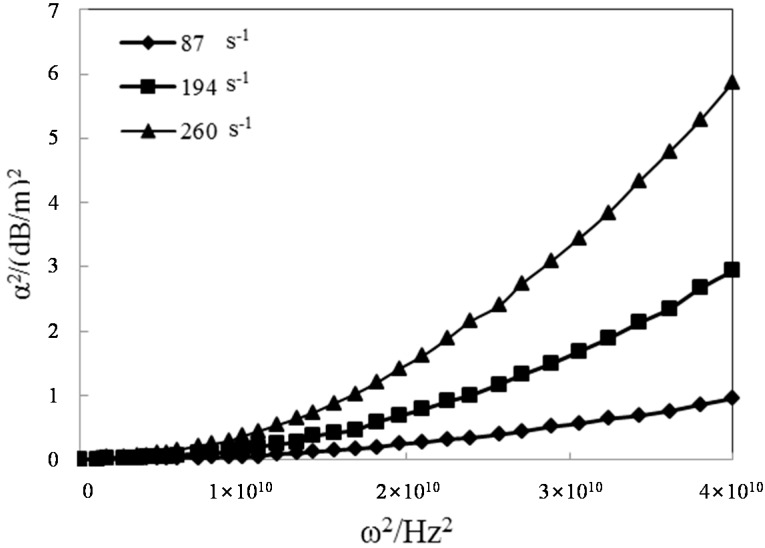
Effects of the stress wave frequency on the attenuation factors in viscoelastic-plasticity.

The relationship between the attenuation factor and the frequency of the stress wave can be explored using the parameters in the constitutive equation of viscoelastic-plastic rock-like materials obtained from the experimental test using the SHPB apparatus. The viscoelasticity formula Equation (20a) and the viscoelasticity plasticity formula Equation (23a) can be used for analysis. As shown in Figure 6 and Figure 7, the square of the attenuation factor is closely correlated with the frequency of the stress wave. An increase in the frequency of the stress wave increases the attenuation factor, regardless of the strain rate.

**Figure 8:**
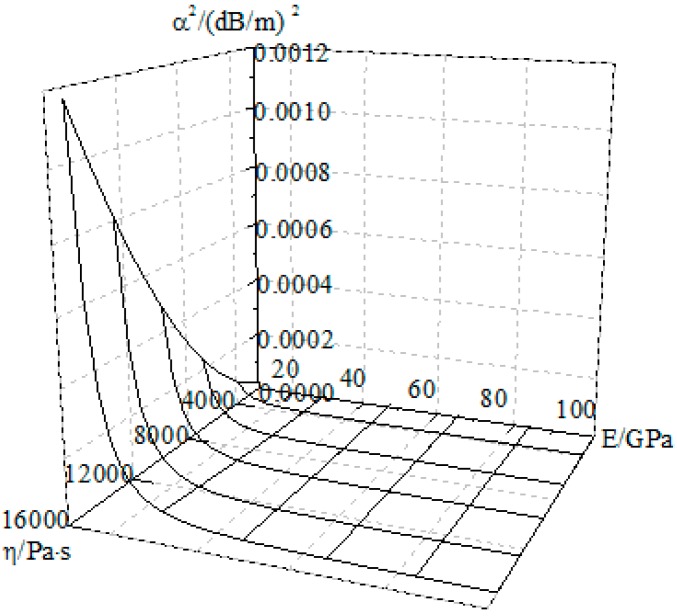
Variations in the attenuation factor with the elastic modulus and the viscosity coefficient.

According to Equation (22), the variations in the attenuation factor with respect to the elastic modulus and the viscosity coefficient were obtained, as shown in Figure 8. Based on the analysis results, the attenuation factor decreased with increasing elastic modulus, whereas it increased with increasing viscosity coefficient. On the other hand, the elastic modulus increased with increasing strain rate, and the viscosity coefficient decreased with increasing elastic modulus. The increase in the attenuation factor decelerates with increasing strain rate if the frequency of the stress wave increases. This suggests that the attenuation factor is a parameter related to the strain rate. An increase in the strain rate reduces the attenuation amplitude of the stress wave. Based on a comparison of Figure 6 and Figure 7, the elastic modulus decreases significantly based on the viscoelastic-plasticity. That is, the attenuation factor is much higher than the viscoelastic damping factor for the three strain rates.

Viscous attenuation induced by the material viscosity is called the absorption phenomenon. Compared to geometric dispersion by slender bar size, the changes in the frequency in viscous attenuation always develops in opposite directions. For the propagation of the stress wave along viscoelastic-plastic rock-like materials, the attenuation of the high-frequency wave is greater than that of the low-frequency wave, and geometric dispersion induced by the lateral inertia of the slender bar is more significant in the low-frequency wave than that in the high-frequency wave.

## 4. Conclusions

This theoretical and experimental study investigated the stress wave propagation in viscoelastic-plastic rock-like materials under a high-strain-rate load and examined the characteristics of strain-dependent attenuation.

Based on element model theory, composite materials, such as rock and concrete, are assumed to be a mixture composed of elastic, plastic, and viscosity components. A constitutive equation of viscoelastic-plastic rock-like materials was developed based on an analytic model composed of an elastomer, a Newtonian body, and a St. Venant body.

Stress wave equations are derived based on the kinematic and kinetic equations considering a nonlinear viscoelastic-plastic constitutive equation. The analytical solution for stress wave propagation in viscoelastic-plastic rock-like materials suggests that the attenuation factor can be determined by the viscous coefficient of the material, the elastic modulus, density, and the frequency of the stress wave.

The analytical solution derived in this study was verified through an experimental test using an SHPB apparatus. Unknown parameters of the constitutive equations were estimated inversely using a differential evolution algorithm. According to the results, the estimated stress-strain curve from the analytic solution is identical to the stress-strain curve obtained from the experimental test, regardless of the strain rate, and the mean square error between them is less than 10%.

Stress wave propagation and the attenuation factor in viscoelastic-plastic materials are affected by the viscous coefficient, the elastic modulus, density, and the frequency of the stress wave. The attenuation factor decreases with increasing elastic modulus, whereas it increases with increasing viscosity coefficient. The attenuation factor increases with increasing frequency of the stress wave. The attenuation of the stress wave is dependent on the strain such that an increase in the strain rate decelerates the increase in attenuation factor.

## Figures and Tables

**Table 1:** Inverse analysis of the parameters in the constitutive equations for viscoelastic-plastic rock-like materials.

Sample Number	Mean Strain Rate (s^−1^)	Estimated Parameters from Inverse Analysis			
		Elastic Modulus (N/m^2^)		Viscosity Coefficient, η	Variance, ${\overset{⌢}{\sigma }}^{2}$
		*E*_1_	*E*_2_		
C01	87	3.499 × 10^10^	3.217 × 10^10^	1.200 × 10^4^	9.077 × 10^−2^
C02	194	3.498 × 10^10^	3.368 × 10^10^	1.093 × 10^4^	8.572 × 10^−3^
C03	260	3.488 × 10^10^	3.457 × 10^10^	8.311 × 10^3^	2.239 × 10^−2^

## References

[1] Follansbee P.S., Frantz C. (1983). Wave propagation in the split Hopkinson pressure bar. J. Eng. Mater. Technol..

[2] Li X.B., Lo T.S., Zhao J., Zhao P.J. (2000). Oscillation elimination in the Hopkinson bar apparatus and resultant complete dynamic stress-strain curve for rocks. Int. J. Rock Mech. Min. Sci..

[3] Mustaqim M.N.M., Song K.I., Cho G.C., Zainab M. (2014). Long-Wavelength Elastic Wave Propagation across Naturally Fractured Rock Masses. Rock Mech. Rock Eng..

[4] Zhai Y., Ma G.W., Zhao J.H. (2007). Dynamic capability of granite and concrete under impact compressive loading. Chin. J. Rock Mech. Eng..

[5] Zhai Y., Zhao J.H., Li X.C. (2011). Study on damage viscoelasticplastic dynamic constitutive model of rock-like materials. Chin. J. Rock Mech. Eng..

[6] Ozbolt J., Sharma A. (2011). Numerical simulation of reinforced concrete beams with different shear reinforcements under dynamic impact loads. Int. J. Impact Eng..

[7] Zhou F.H., Chen L., Wang L.L. (2010). Two approaches for analyzing one-dimensional viscoelastic wave propagations. Eng. Mech..

[8] Graham G.A.C., Sabin G.C.W. (1973). The correspondence principle of linear viscoelasticity for problems that involve time-dependent regions. Int. J. Eng. Sci..

[9] Raymond S., Lemiale V., Ibrahim R., Lau R. (2014). A mesh free study of the Kalthoff-Winkler experiment in 3D at room and low temperatures under dynamic loading using viscoplasticmodeling. Eng. Anal. Bound. Elem..

[10] Liu H.S., Tang L., Bo J.S., Feng Z. (2009). Elastic-viscoplastic analysis of explicit FEM for simulating wave motions in rock mass. Coal Geol. Explor..

[11] Passaris E.K.S. The rheological behavior of rock salt as determined in an *in situ* pressure test cavity. Proceedings of the 4th Conference on International Society for Rock Mechanics.

[12] Ding J.Y., Zhou H.W., Liu D. (2014). Research on fractional derivative three elements model of salt rock. Chin. J. Rock Mech. Eng..

[13] Xie L., Zhao G.M., Meng X.R. (2014). Research on damage viscoelastic dynamic constitutive model of soft rock and concrete materials. Chin. J. Rock Mech. Eng..

[14] Liu S., Xu J.Y., Chen T.F., Wang P. (2013). Study on dynamic response of rock based on split Hopkinson pressure bar test. Chin. J. Undergr. Space Eng..

[15] Yan S.H., Duan J.X., Yin F.L., Qian Q.H. (2000). SHPB test on high-strength concrete. J. PLA Univ. Sci. Technol..

[16] Zhao H., Gray G. (1996). On the use of SHPB techniques to determine the dynamic behavior of materials in the range of small strains. Int. J. Solids Struct..

[17] Hertlein B.H. (2013). Stress wave testing of concrete: A 25-year review and a peek into the future. Construct. Build. Mater..

[18] Wang L.L. (2005). Foundations of Stress Waves.

[19] Li Z.Q., Chen W.Y., Wang Z.H. (2010). Study on the effect of striker bar length on the SHPB measurements. J. Mech. Strength.

[20] Liu L., Song K.I., Lao D.Z., Kwon T.H. (2015). Rheological properties of cemented tailing backfill and the construction of a prediction. Model. Mater..

[21] Zhou J., Li X.B., Shi X.Z. (2012). Long-term prediction model of rockburst in underground openings using heuristic algorithms and support vector machines. Saf. Sci..

[22] Li J.G., Cheng G.Q., Zou Y.H. (2008). Study on the Stress Wave Propagation Attenuation Laws in the Elastoplastic Coal and Rock. Master’s Thesis.

